# Inclusivity in prostate cancer and exercise research: a systematic review

**DOI:** 10.1007/s00520-024-08793-9

**Published:** 2024-08-28

**Authors:** Ruth E. Ashton, Mark A. Faghy, Clare M. P. Roscoe, Jonathan Aning

**Affiliations:** 1 Research Centre for Physical Activity, Sport and Exercise Sciences, Coventry University, Coventry, UK; 2 Biomedical and Clinical Science Research Theme, School of Human Sciences, University of Derby, Derby, UK; 3 Healthy Living for Pandemic Event Protection (HL – PIVOT) Network, Chicago, IL USA; 4 Department of Physical Therapy, College of Applied Sciences, University of Illinois at Chicago, Chicago, IL USA; 5 Bristol Urological Institute, North Bristol NHS Trust, Southmead Hospital, Southmead Road, Westbury-On-Trym, Bristol, UK; 6 Population Health Sciences, Bristol Medical School, University of Bristol, Bristol, UK

**Keywords:** Prostate cancer, Exercise, Inclusivity, Ethnic diversity

## Abstract

**Background:**

Prostate cancer (PCa) is the most prevalent type of cancer in men in the UK. Exercise has been shown to improve the health and quality of life of PCa patients. Exercise should be easily accessible to men with PCa regardless of socioeconomic group or ethnicity. There is a need to better understand whether the current evidence base for exercise interventions is representative and inclusive of racial and ethnic minority men with PCa.

**Methods:**

A systematic review of the literature was conducted according to PRISMA guidelines and prospectively registered via Prospero (ID: CRD42022384373). The MEDLINE Ovid, Cochrane Library and PubMed databases were searched from inception to December 2022. The search strategy keywords and MeSH terms used included the following: (1) exercise, (2) training, (3) prostate cancer, (4) ethnic and (5) diversity.

**Results:**

A total of 778 records were retrieved from database searches, of which 15 records were duplicates. A further 649 were eliminated following the screening of titles and abstracts. After full-text screening of 186 articles, 28 manuscripts were included for review.

**Conclusion:**

This systematic review highlights that there is high heterogeneity in the reporting of participants’ ethnicity and there are low numbers of ethnic minority men included in PCa and exercise studies in the UK. Further work is required to understand why representation is lacking within PCa exercise trials in the UK and strategies are needed to achieve representation from all ethnic groups.

**Implications for cancer survivors:**

Improved representation and reporting of ethnicity in exercise trials is vital to ensure the results are applicable to all patients.

## Background

Prostate cancer (PCa) is the most prevalent type of cancer in men in the UK, with approximately 55,100 confirmed new cases each year and a further 1.4 million worldwide [1, 2]. Effective treatments for localised and metastatic PCa are recognised to have side effects which may be associated with negative impacts on patients’ quality of life, functional status and physical and mental health [3–6]. Despite advances in PCa management and improvements in outcomes driven by clinical trials, it is recognised that significant PCa health disparity remains due to a poorly understood, complex interplay of factors [7]. It is well documented that Black men are at a 2.1 times greater risk of being diagnosed with PCa (23.5–37.2%) compared to individuals of White (13.2–15.0%) or Asian (6.3–10.5%) ethnicity [8, 9]. Black men are more likely to be diagnosed at a younger age and with a more advanced stage PCa compared to men of other ethnicities. Advanced PCa can have a significant impact on patients’ survival, quality of life and ability to carry out activities of daily living effectively [10].

Exercise has been demonstrated to improve the health outcomes and quality of life of men with PCa [11–13]. The evidence supporting the benefits of exercise in men with PCa has become more compelling over the past decade, to the extent that exercise is recommended in current national and international PCa guidelines [14, 15]. The effect of aerobic, resistance and flexibility exercise on patients’ outcomes at different stages of the pathway and in multiple settings for example home, community and hospital has been researched over varying durations [16, 17]. Exercise during and after PCa treatment is safe and effective at improving important health outcomes, including improved aerobic capacity and body composition [6, 11, 18]. Varying exercise modalities have been shown to improve body composition, strength, blood pressure, blood biomarkers such as plasma triglycerides and fasting plasma glucose, aerobic capacity, quality of life and attenuate fatigue [11, 12, 19–21]. Interventions involving combined exercise modalities, such as aerobic and strength training concurrently, have been demonstrated to improve bone density [6].

Exercise opportunities should be easily accessible to men with PCa regardless of racial or ethnic minority status. Recent reports have highlighted that diversity and representation are poor in PCa clinical trials, with approximately 96% of men participating in PCa research being White [22]. There is a need to understand whether the present evidence base for exercise interventions is representative and inclusive of racial and ethnic minority men with PCa and, if not, explore the barriers and seek tailored acceptable interventions or strategies which are more inclusive. The aim of this systematic review is to examine current reporting of race and ethnicity data and strategies to increase inclusivity and demographic representation within PCa and exercise trials.

## Methods

### Protocol and registration

The preferred reporting items for systematic review and meta-analyses (PRISMA) guidelines were followed when conducting and reporting this prospectively registered systematic review (PROSPERO ID: CRD42022384373) [23].

### Eligibility criteria

We included trials published in the English language that studied exercise interventions in PCa patients. Manuscripts were excluded if they were protocols, reports, conference abstracts, position statements or case series reports. Participants must have been aged > 16 years and have been diagnosed with PCa. Studies could include any form of exercise intervention in isolation or combined with other lifestyle factors such as nutritional supplementation or smoking cessation. Studies that included either details of participants’ ethnicity or strategies to ensure inclusion and representation were eligible.

### Search strategy

The MEDLINE Ovid, Cochrane Library and PubMed databases were searched from inception to July 2024. The search strategy keywords and MeSH terms used included the following: (1) exercise, (2) training, (3) prostate cancer, (4) ethnic and (5) diversity. Reference lists of all relevant systematic reviews identified were searched for additional studies. All searches were conducted by the same author (RA), with search results collated using Rayyan software [24], and duplicates were removed. All titles, abstracts and full texts were screened by one reviewer (RA). Any uncertainty of a manuscript was resolved by a second reviewer (MF).

### Data extraction

One author (RA) extracted data using Microsoft Excel which was checked by a second author (MF). Any disagreements were resolved via consensus with a third party (CR). Extracted data included study design, participant demographics, intervention details and data for all outcomes.

## Results

**Fig. 1:**
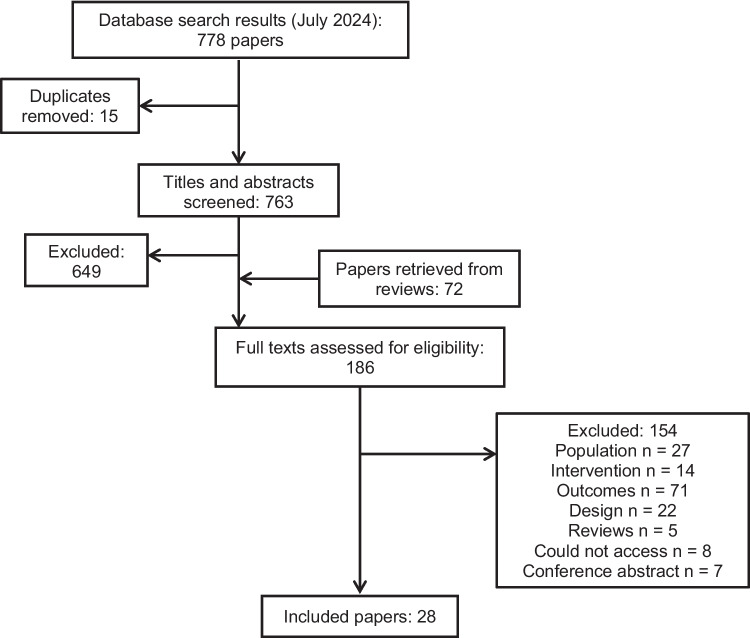
PRISMA flow diagram

**Fig. 2:**
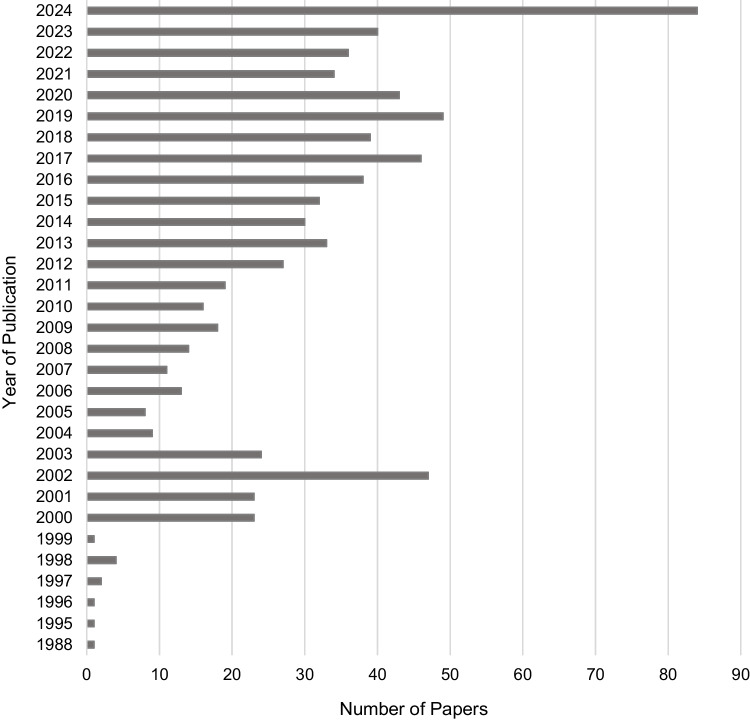
Volume of peer-reviewed academic articles published per year included in the screening

Seven hundred and seventy-eight records were retrieved from database searches, of which 15 records were duplicates. A further 649 were eliminated following a screening of titles and abstracts. After the full-text screening of 186 articles, 28 manuscripts were found to be eligible for inclusion in this review (Fig. 1) with an increase in the volume of papers published over the last three decades (Fig. 2).

### Characteristics of studies

The 28 studies included comprised 15,946 participants, with sample sizes ranging from 11 to 7328 per study. Summary details of the included studies and populations are presented in Table 1.

**Table 1:** Characteristics of included studies

Authors	Title	Country	Setting	Number of centres	Study Design	Treatment	Trial enrolment duration
Alibhai et al. [25]	A Phase II RCT of Three Exercise Delivery Methods in Men with PCa on ADT	CAN	Secondary, home	Multiple	RCT	ADT	6 months
Ashton et al. [11]	Supported Progressive Resistance Exercise Training to Counter the Adverse Side Effects of Robot-Assisted RP: An RCT	UK	Home	Single	RCT	RP	24 weeks
Au et al. [26]	Prehabilitation and Acute Postoperative Physical Activity in Patients Undergoing RP: A Secondary Analysis from An RCT	CAN	Home	Multiple	RCT	RP	Post-op day 1 until 7 days post-discharge
Ben-Josef et al. [27]	Yoga Intervention for Patients with PCa Undergoing External Beam RT: A Pilot Feasibility Study	USA	Secondary	Single	Feasibility	RT	6–9 weeks dependent on the RT course
Brown et al. [28]	Feasibility of home‑based exercise training during adjuvant treatment for metastatic castrate‑resistant prostate cancer patients treated with an androgen receptor pathway inhibitor (EXACT)	UK	Home	Single	Feasibility	ADT	12 weeks
Campo et al. [29]	Levels of Fatigue and Distress in Senior PCa Survivors Enrolled in a 12-Week RCT of Qigong	USA	Secondary, home	Single	RCT	Any	12 weeks
Chan et al. [30]	Feasibility and Acceptability of a Remotely Delivered, Web-Based Behavioural Intervention for Men with PCa: Four-Arm Pilot RCT	USA	Home	Multiple	RCT	Any	6 months
Dai et al. [31]	Vigorous Physical Activity is Associated with Lower Risk of Metastatic-Lethal Progression in PCa and Hypomethylation in the *CRACR2A* Gene	USA	Not stated	Not stated	Cohort	Any	Not stated
Dalla Via et al. [32]	Musculoskeletal Responses to Exercise Plus Nutrition in Men with PCa on ADT: A 12-Month RCT	AUS	Home, community	Multiple	RCT	ADT	12 months
Dawson et al. [33]	Impact of Resistance Training on Body Composition and Metabolic Syndrome Variables During ADT for PCa: A Pilot RCT	USA	Home, community	Not stated	RCT	ADT	12 weeks
Evans et al. [34]	Usability, Acceptability, and Safety Analysis of a Computer-Tailored Web-Based Exercise Intervention (Exercise Guide) for Individuals with Metastatic PCa: Multi-Methods Laboratory-Based Study	AUS	University	Not stated	Laboratory-based assessment	Any	Not stated
Fairman et al. [35]	Monitoring Resistance Exercise Intensity Using Ratings of Perceived Exertion in Previously Untrained Patients with PCa Undergoing ADT	USA	University	Single	Secondary analysis	ADT	Not stated
Faithfull et al. [36]	Obesity and Low Levels of Physical Activity Impact on Cardiopulmonary Fitness in Older Men After Treatment for PCa	UK	Secondary, university	Multiple	Cross-sectional	RP, RT or ADT	Single visit
Friedenreich et al. [37]	Physical Activity and Survival After PCa	CAN	Secondary, university	Multiple	Case–control	Any	14 years
Gilbert et al. [38]	Effects of a Lifestyle Intervention on Endothelial Function in Men on Long-Term ADT for PCa	UK	Home, community	Not stated	RCT	ADT	12 weeks
Hébert et al. [39]	A Diet, Physical Activity, and Stress Reduction Intervention in Men with Rising Prostate-Specific Antigen After Treatment for PCa	USA	Secondary	Multiple	RCT	RP, RT	6 months
Jones et al. [40]	Effects of Nonlinear Aerobic Training on Erectile Dysfunction and Cardiovascular Function Following RP for Clinically Localized PCa	CAN	Secondary	Single	RCT	RP	6 months
Langelier et al. [41]	Perceptions of Masculinity and Body Image in Men with PCa: The Role of Exercise	CAN	University	Single	Cross-sectional	Any	Not stated
Nilsen et al. [42]	Novel Methods for Reporting of Exercise Dose and Adherence: An Exploratory Analysis	USA	Secondary	Single	RCT	RP	24 weeks
Sajid et al. [43]	Novel Physical Activity Interventions for Older Patients with PCa on Hormone Therapy: A Pilot Randomized Study	USA	Home	Not stated	RCT	ADT	12 weeks
Santa Mina et al. [44]	Exercise Effects on Adipokines and The IGF Axis in Men with PCa Treated with ADT: A Randomized Study	CAN	Home	Single	Prospective, randomised trial	ADT	24 weeks
Santa Mina et al. [45]	Prehabilitation for RP: A Multicentre RCT	CAN	Home	Multiple	RCT	RP	26 weeks
Stolley et al. [46]	Exploring Health Behaviours, Quality of Life, and Support Needs in African-American PCa Survivors: A Pilot Study to Support Future Interventions	USA	Community	Single	Cross-sectional	Any	Not stated
Taaffe et al. [47]	Effects of Different Exercise Modalities on Fatigue in PCa Patients Undergoing ADT: A Year-long RCT	AUS	University, community	Multiple	RCT	ADT	12 months
Trinh et al. [48]	RiseTx: Testing the Feasibility of a Web Application for Reducing Sedentary Behaviour Among PCa Survivors Receiving ADT	CAN	Secondary, home	Multiple	Prospective, single-arm	ADT	12 weeks
Wang et al. [49]	Recreational Physical Activity in Relation to PCa-specific Mortality Among Men with Nonmetastatic PCa	USA	Home	Multiple	Cohort	Any	Not stated
Winters-Stone et al. [50]	Benefits Of Partnered Strength Training for PCa Survivors and Spouses: Results from A RCT of The Exercising Together Project	USA	University	Single	RCT	Any	6 months
Wolin et al. [51]	Risk of Urinary Incontinence Following RP: The Role of Physical Activity and Obesity	USA	Secondary	Single	Cross-sectional	RP	50–74 weeks

*CAN* Canada, *UK* United Kingdom, *AUS* Australia, *USA* United States of America, *PCa* prostate cancer, *RCT* randomised control trial, *ADT* androgen deprivation therapy, *RT* radiotherapy, *RP* radical prostatectomy

The majority of studies (21/28) were performed in North America. Eleven studies (12/28) were conducted at a single site and another 11/28 recruited patients from multiple sites with the remaining 5/28 not stating the number of recruiting sites. The trial enrolment duration varied from 1 day to 14 years with 3/28 studies stating a range of lengths depending on the patient and 6/28 studies not stating the trial duration.

### Narrative synthesis

All studies included reported the ethnicity of participants. Four studies 4/28 (14.3%; [28, 34, 44, 47]) only reported including Caucasian individuals and one study 1/28 (3.6%; [46]) reported only including African American patients. The remaining studies 23/28 (82.1%; [11, 25–27, 29–33, 35–43, 45]) all recruited patients from multiple ethnicities. No studies included a statement regarding inclusivity as part of the published manuscripts.

Fifteen studies 15/28 (53.6%; [11, 25, 26, 29–31, 33, 38–40, 42, 43, 45, 47, 50]) adopted a randomised control trial design, four 4/28 (14.3%; [36, 41, 46, 51]) cross-sectional, two 2/28 (7.1%; [31, 49]) cohort, two prospective 2/28 (7.1%; [44, 48]), two feasibility 2/28 (7.1%; [27, 28]), laboratory analysis 1/28 (3.6%; [34]), secondary analysis 1/28 (3.6%; [35]) and case–control study 1/28 (3.6%; [37]). Ten studies 10/28 (35.7%; [25, 28, 32, 33, 35, 38, 43, 44, 47, 48]) only recruited patients on androgen deprivation therapy (ADT), nine studies 9/28 (32.1%; [29–31, 34, 37, 41, 46, 49, 50]) involved patients on any form of treatment and six studies 6/28 (21.4%; [11, 26, 40, 42, 45, 51]) recruited those who had undergone radical prostatectomy. One study 1/28 (3.6%; [27]) recruited those receiving radiotherapy only, one study 1/28 (3.6%; [39]) recruited either radical prostatectomy or radiotherapy and a single study 1/28 (3.6%; [36]) involved either ADT, radical prostatectomy or radiotherapy patients.

The exercise elements of the studies varied, and individual study exercise details are presented in Table 2. Seven studies 7/28 (25%; [26, 28, 30, 43–45, 49]) involved home-based or unsupervised exercise. Six studies 6/28 (21.4%; [27, 29, 34, 40, 42, 50]) reported exercise sessions that were supervised by an exercise trainer or exercise physiologist and a further six studies 6/28 (21.4%; [11, 25, 32, 33, 38, 47]) had a mix of supervision over the trial period. Nine studies 9/28 (32.1%; [31, 35–37, 39, 41, 46, 48, 51]) did not explicitly state whether exercise was supervised or unsupervised. Three studies 3/28 (10.7%; [11, 35, 50]) included resistance exercise interventions, two 2/28 (7.1%; [40, 42]) included aerobic exercise interventions and a further two studies 2/28 (7.1%; [27, 29]) included yoga or flexibility programmes. Eleven studies 11/28 (39.3%; [25, 26, 28, 32, 33, 36, 38, 43–45, 47]) investigated an intervention which used a mix of exercise modalities (i.e. aerobic, resistance and flexibility). Ten studies 10/28 (35.7%; [30, 31, 34, 37, 39, 41, 46, 48, 49, 51]) were physical activity-based and primarily involved activity tracking.

**Table 2:** Intervention and ethnicity data from included studies

**Authors**	**Age** (mean ± SD)	**Exercise details**	**Total sample size**	**Ethnicity**
Alibhai et al. (2019)	PT = 69.2 ± 7.3 Group = 71.5 ± 7.2 HOME = 69.6 ± 8.1	Three exercise delivery arms: (1) 1:1 supervised training (PT), (2) supervised group training (GROUP), (3) home-based smartphone-assisted training (HOME). 4–5 days per week of mixed modality exercise incorporating AET, RET and flexibility. A relative workload of 60–70% HR reserve was consistent across groups. The intensity was monitored using the 10-point RPE scale	65	White (71.7%) Other (28.3%)
Ashton et al. (2021)	EX = 64.6 ± 6.2 CON = 66.9 ± 6.8	Three weekly sessions of resistance band RET. 3 sets of 12–15 reps for 8–10 exercises targeting the major muscle groups. Exercises were performed with 30–60 s interpolated rest intervals until 3 sets of each exercise had been performed. Tapered supervision over the first 3 months	42	White British (97.6%) Other (2.4%)
Au et al. (2019)	EX = 61.4 ± 7.8 CON = 58.4 ± 6.1	Individualized, home-based, moderate-intensity AET and RET prescribed. Provided with exercise bands, an exercise mat and a stability ball to complete their program, in addition to a manual detailing their exercise prescription with supporting behaviour change strategies. Information and coaching on pelvic floor exercises to complete prior to surgery targeting earlier recovery of urinary control after surgery	86	White/Caucasian (71.1%) Black/Afro-Caribbean/African (13.2%) Ashkenazi Jewish (2.6%) East and South Asian (7.9%) Arabic (2.6%) Hispanic (2.6%)
Ben-Josef et al. (2016)	*Mean (range)* 66.4 (51–74)	Classes were led by a trained instructor and lasted 75 min. A typical session included seated, standing and reclining poses. Yoga poses were modified and included the use of props to facilitate and adapt the poses for each participant. Sessions began with breathing and centring techniques	15 (45 recruited)	White (68.9%) Black (24.4%) Asian (6.7%)
Brown et al. (2023)	71 ± 6	Home-based intervention of progressive, moderate-intensity walking and RET, 2–5 times per week. RET was performed using body mass and dumbbells (or weighted household items depending on dumbbell accessibility). Participants were provided with a pedometer (Digi-Walker, Yamax) to determine step count during exercise, an exercise booklet to log sessions and RPE	22 (30 recruited)	White (100%)
Campo et al. (2014)	*Median (range)* EX = 72 (58–90) UC = 73 (61–93)	The Qigong and non-aerobic stretching (UC) exercise classes were 60 min and held twice a week. Both groups received a DVD of progressive sessions of each intervention. Qigong classes were led by a certified instructor. Sessions began with a 5-min meditative focus on the breath, followed by sitting and standing exercises, and ended with a 5-min meditative focus on the breath. The study progressed with more time spent performing the standing exercises	40	Non-Latino (96.6%) Latino (3.4%) White (93.1%) Non-white (6.9%)
Chan et al. (2020)	*Median (IQR)* Level 1 = 70 (64–76) Level 2 = 70 (64–75) Level 3 = 70 (64–75) Level 4 = 70 (65–74) Total = 70 (65–75)	Level 1 (reference group): received general information about exercise and diet and resources Level 2: same as level 1 and a personalised diet and exercise prescription, videos of recommended exercises and a weekly survey to track progress Level 3: same as level 2 and a Fitbit Alta with PA reports, supportive text messages and weekly web-based surveys to track progress Level 4: same as levels 3 and 2 optional 30-min calls with an exercise trainer/registered dietician	202	White (92.6%) Black (2.5%) Asian (1.0%) Other (0.5%) More than one race (2.5%) Decline to answer (1.0%)
Dai et al. (2019)	*Median* < 1 h per week = 60 1–3 h per week = 59 > 3 h per week = 60	Vigorous PA was defined to be any type of leisure time activity that lasts more than 20 min or works up for a sweat in the questionnaire. Data collected on the number of days in a week having vigorous PA in the year pre-diagnosis were analysed. Light and moderate PA data was not collected. Men were grouped into three categories of vigorous PA frequency in this analysis—greater than 3 times a week, 1–3 times a week or < 1 time per week vigorous PA	1354	Caucasian (90.0%) African American (10.0%)
Dalla Via et al. (2021)	EX = 71.4 ± 5.9 CON = 71.1 ± 6.6	Two gym-based sessions of AET, 5–6 RET exercises (2 sets, 8–12 reps at mod-hard intensity), three weight-bearing impact exercises (3 sets, 10–20 reps), two challenging balance/functional exercises (2 sets of 30–60 s) and two core stability exercises (2 sets, 10–15 reps). During the first 6 months, two weekly sessions were supervised by an exercise physiologist reduced to one. One weekly home-based session (20–60 min) using body weight and resistance bands. One sachet of a multi-nutrient supplement (powder mixed with 150 mL of water) and a vitamin D tablet was taken daily in addition to regular diet	70	Caucasian (97.2%) Asian (1.4%) African (1.4%)
Dawson et al. (2018)	TRAINPRO AND TRAIN = 68.6 ± 8.4 PRO AND STRETCH = 66.3 ± 9.0	TRAINPRO and TRAIN groups performed RET 3 days per week with a trainer. Sessions were ~ 50 min in duration and began with a 5-min warmup. Weekly training volume was divided so each muscle group was trained twice per week. PRO and STRETCH groups performed a home-based flexibility program 3 times per week. Each session matched the stretches performed by the TRAIN and TRAINPRO groups. PRO and STRETCH groups acted as CON and were given a stretching band and booklet detailing the exercises	35	White (54.3%) African American (8.6%) Asian/Pacific Islander (25.7%) Hispanic (11.4%)
Evans et al. (2021)	73.37 ± 6.7	PA behaviour was measured using the modified Godin Leisure-Time Exercise Questionnaire. The weekly frequencies (longer than 15 min) of vigorous, moderate and light physical activities were weighted and summed to obtain a total score in units	11	Caucasian (100%)
Fairman et al. (2018)	68.8 ± 9.07	1RM testing for chest press and leg extension after completing a warm-up set of 10–12 reps with roughly 10–20% of body weight, depending on patient characteristics and previous experience. Participants were asked to lift the weight once and to continue to perform single rep lifts with increasing weight, separated by a 3–5-min rest, until a max weight was reached	77	White (92.2%) African American (6.5%) Asian (1.3%)
Faithfull et al. (2021)	68.2 ± 7.4	Grip strength measured upper body strength. A 30-s chair sit-to-stand time measured lower body strength. CPET pedalling frequency was self-selected within a given range. After a 2-min warm-up against no resistance, the intensity increased to 20–30 Watts/min. Men were encouraged to continue cycling to volitional exhaustion or a plateau in VO2	83	Caucasian (96.4%) Black British (3.6%)
Friedenreich et al. (2016)	Not stated	Interviews on lifetime PA were completed 4.3 ± 1.3 months post-diagnosis. The Lifetime Total Physical Activity Questionnaire was from childhood until diagnosis. Diet was reported for the year pre-diagnosis and height and weight for each decade of 20–60 years. Post-diagnosis PA was measured up to three times per participant using interviews and mail questionnaires	830	White (95.0%) Other (5.0%)
Gilbert et al. (2016)	EX = 70.1 ± 5.3 CON = 70.4 ± 9.2	Three sessions per week led by an exercise physiologist, tapering supervision over time. Sessions consisted of AET, RET and balance exercises. AET 30 min at 55–75% of age-predicted max HR or 11–13 RPE using ergometers and treadmills. RET 2–4 sets of 8–12 reps beginning at 60% 1RM. Advice on home exercise was provided. Healthy-eating seminars ~ 20 min delivered biweekly	50	White (94.0%) Asian (4.0%) Black (2.0%)
Hébert et al. (2012)	EX = 69.7 ± 8.8 CON = 71.1 ± 8.1	A single session where dietary and PA goals were discussed and set as well as 2.5-h group sessions conducted 3 times a week for the first 3 months. Monthly group booster sessions and progress calls continued for 3 months after. Participants were given daily ‘homework’ assignments that consisted of cooking, PA and stress reduction activities	60 (47 analysed)	White/European American (70.2%) Black/African American (29.8%)
Jones et al. (2014)	EX = 58 ± 7 CON = 61 ± 5	AET of 72 supervised treadmill walking sessions 3 days a week. The intensity of each session alternated between five different doses of MET expenditure (i.e. VO2peak). The intensity was individualized to each patient based on workload (i.e. treadmill speed/grade) corresponding to a specific percent of VO2peak	50	White (70.0%) Black (26.0%) Asian (4.0%)
Langelier et al. (2018)	65.5 ± 8.5	Questionnaires assessing demographic information, masculine values, body image, QoL and PA levels. The Godin’s Leisure Score Index of the Godin and Shephard Leisure Time Exercise Questionnaire was used to assess current PA levels	50	Non-Hispanic White/Euro American (92.0%) Latino/Hispanic American (4.0%) East Asian/Indian American (2.0%) Middle Eastern/Arab American (2.0%)
Nilsen et al. (2018)	EX = 58 ± 8 CON = 61 ± 5	AET of 72 supervised treadmill walking sessions 3 days a week. The intensity of each session alternated between five different doses of MET expenditure (i.e. VO2peak). The intensity was individualized to each patient based on workload (i.e. treadmill speed/grade) corresponding to a specific percent of VO2peak directly measured during the baseline or midpoint CPET	50	White (76.0%) Black (24.0%) Asian (0.0%)
Sajid et al. (2016)	Wii-Fit = 77.5 ± 6.7 EXCAP = 75.7 ± 9.5 CON = 71.8 ± 5	EXCAP: AET was walking at 60–70% of HR reserve and 3–5 RPE 5 days a week. Instructed to increase total daily steps by 5% and were encouraged to reach 10,000 steps a day using a pedometer. Progressive RET with bands provided low-moderate intensity 5 days a week and progressed. Wii-Fit: similar to EXCAP with a balance component and pedometer to calculate daily steps. Exercises of increasing intensity were unlocked as patients increased physical performance	19	White (87.5%) African American (12.5%)
Santa Mina et al. (2013)	AET = 70.6 ± 8.1 RET = 73.6 ± 8.8	RET group completed 10 exercises targeting major muscle groups using resistance bands, exercise mat and stability ball. Exercised 5 times per week for 60 min. AET exercised at moderate to vigorous intensity (60- 80% HR max) using a HR monitor provided. Exercised 5 times per week for 60 min	26	Caucasian (65.4%)
Santa Mina et al. (2018)	EX = 61.2 ± 8.0 CON = 62.2 ± 6.9	Exercise prescriptions consisted of 60 min of unsupervised, home-based, moderate-intensity exercise 3–4 days per week. Also received an exercise manual, online videos, RET bands, stability ball, yoga mat and HR monitor. Completed daily pelvic floor muscle exercises. CON received pelvic floor exercises and lifestyle manual	86	White/Caucasian (72.3%) Black/Afro-Caribbean/African (13.3%) Ashkenazi Jewish (1.2%) East and South Asian (4.8%) Southeast Asian (1.2%) Other (6.0%) Missing (1.2%)
Stolley et al. (2020)	64.3 ± 4.0	Completion of questionnaires on demographics, nutrition, physical activity patterns and QoL. Discussions focused on health behaviour change/needs, interests and preferences of lifestyle intervention. Godin Leisure Physical Activity Index asked about time spent engaged in light, moderate and strenuous PA over the past 7 days and engagement in RET per week	22	African American (100.0%)
Taaffe et al. (2017)	ILRT = 68.9 ± 9.1 ART = 69.0 ± 9.3 DEL = 68.4 ± 9.1	ILRT: twice weekly supervised sessions of bounding/skipping/drop jumping/hopping/leaping. RET consisted of six exercises targeting major muscle groups. 2–4 sets of each exercise at 6–12RM. Home training twice weekly consisting of 2–4 circuits of skipping/hopping/leaping ART: twice weekly supervised sessions for the first 6 months. AET consisted of 20–30 min at 60–75% of estimated max HR using walking/jogging and stationary ergometers. RET during the initial 6 months was the same as the ILRT group. Encouraged to undertake home-based AET (e.g., walking/cycling) to accumulate 150 min/week. For the second 6 months, patients completed a home-based maintenance program UC/DEL received an information booklet about exercise for the first 6 months, followed by 6 months of twice weekly supervised exercise on a cycle ergometer at ~ 70% max HR and flexibility exercises	159	Caucasian (not stated)
Trinh et al. (2018)	73.2 ± 7.3	A wrist-worn activity tracker providing alerts to stand after prolonged sitting. The intervention consisted of five phases. Phases I-III (weeks 3–6) involved the progressive release of self-regulatory strategies (e.g., action planning) and changes in sitting time and step counts. Phase IV and V (weeks 9–12) received weekly reminders to encourage the use of RiseTx to practice Phases I–III strategies. Participants attempted to increase daily steps by + 1000 step increments above the previous phase	46	White (80.4%) Black (8.7%) South Asian (4.3%) Southeast Asian (2.2%) Other (4.3%)
Wang et al. (2017)	*Median (IQR) at diagnosis* 71 (67–75)	Recreational PA per week during the past year was self-reported on the baseline questionnaire and on biennial follow-up questionnaires. METs were assigned to each of the seven activities as follows: 3.5 for walking, 3.5 for dancing, 4.0 for bicycling, 4.5 for aerobics, 6.0 for tennis or racquetball, 7.0 for jogging/running and 7.0 for lap swimming	7328 pre-diagnosis, 5319 post-diagnosis	White (97.4%) Black (1.5%) Other/unknown (1.1%)
Winters-Stone et al. (2016)	Prostate cancer patient: EX = 70.6 ± 6.3 CON = 72.9 ± 8.0 Spouse: EX = 66.5 ± 7.2 CON = 69.7 ± 7.7	Couples assigned to Exercising Together (EX) attended 1-h group sessions twice a week delivered by an exercise physiologist. 5-min dynamic AET warm-up and 5–10 min stretching cool-down. RET 8–10 exercises with 8–15 reps of an exercise at intensities that progressed from 4 to 15% of body weight in a weighted vest for lower body and from a weight that could be lifted for 15 reps to a heavier weight that could be lifted for 8 reps for upper body exercises using free weights	62 couples	Caucasian (92.5%) Non-Hispanic (94.8%)
Wolin et al. (2010)	*Mean (range)* 61 (39–79)	Questionnaire on medical history and lifestyle factors including hours spent on vigorous activities (e.g., swimming, brisk walking). Urinary incontinence data were extracted at the first post-op visit at approximately 6 weeks (range 3 to 17) and at 58 weeks (range 50 to 74). Height and weight at surgery were used to calculate BMI	589	White (95.0%) African American (4.0%) Hispanic (1.0%)

*SD* standard deviation, *EX* exercise group, *CON* control group, *AET* aerobic exercise training, *RET* resistance exercise training, *HR* heart rate, *IQR* interquartile range, *UC* usual care group, *RPE* rating of perceived exertion, *reps* repetitions, *s* seconds, *min* minutes, *UC* usual care, *PA* physical activity, *1RM* one repetition maximum, *CPET* cardiopulmonary exercise test, *VO2* aerobic capacity, *MET* metabolic equivalent, *QoL* quality of life, *ILRT* impact loading and resistance training, *ART* aerobic and resistance training, *DEL* delayed exercise

### Risk of bias

Risk of bias was assessed on all randomised studies and conducted by one author (RA). The Cochrane Risk of Bias tool [52] was used with risk of bias on the study level classified as ‘low’, ‘unclear’ or ‘high’ risk [53]. Table 3 shows a summary of the risk of bias for each of the included studies.

**Table 3:** Risk of bias

Authors	Bias arising from the randomization process	Bias arising from deviations from the intervention	Bias due to missing data	Bias in measurement of outcome	Bias in selection of reported result	Overall risk of bias
Alibhai et al. (2019)	-	-	-	-	?	?
Ashton et al. (2021)	-	-	-	?	?	?
Au et al. (2019)	-	?	?	?	?	?
Campo et al. (2014)	?	?	-	-	?	?
Chan et al. (2020)	-	?	-	-	?	?
Dalla Via et al. (2021)	-	?	-	?	?	?
Dawson et al. (2018)	-	?	-	?	?	?
Fairman et al. (2018)	?	?	?	?	?	?
Gilbert et al. (2016)	-	?	-	-	?	?
Hébert et al. (2021)	?	?	-	?	?	?
Jones et al. (2014)	?	?	-	-	?	?
Nilsen et al. (2018)	-	-	-	?	?	?
Sajid et al. (2016)	-	-	-	-	?	?
Santa Mina et al. (2013)	-	?	-	?	?	?
Santa Mina et al. (2018)	-	?	-	?	?	?
Taaffe et al. (2017)	-	?	-	?	?	?
Winters-Stone et al. (2016)	-	-	-	-	?	?

+, high risk; *?*, unclear risk; *-*, low risk

## Discussion

### Summary of findings

This review highlights the lack of reporting of patient ethnicity in PCa clinical trials involving exercise. Only 28 manuscripts reported ethnicity and none of the studies included a statement regarding strategies for ensuring inclusion or representative sampling. For example, studies may want to consider who the under-served groups are within the delivery area and barriers they face, plan for digital exclusion, recruit a sample who represents those who live with the specific condition, use language carefully or involve those under-served in the planning phase [54]. In the papers included in this review, it is evident that there is inconsistency in how ethnicity is reported making the results of the studies difficult to apply to the general PCa population. Accordingly, there is a greater need for those developing and running clinical trials in PCa and subsequently academic research outputs to adopt standardised terminology when it comes to describing and reporting the race and ethnicity of participants in exercise research, for example using those listed on countries official websites [55, 56]. To the authors’ knowledge, this is the first systematic review investigating ethnicity reporting in PCa and exercise trials and it therefore acts as a baseline for future practice.

Only one pilot trial has been included in this review that specifically recruited black men and this was in an African American community population. This mixed-methods study explored the quality of life, dietary and physical activity habits of African American PCa survivors [46]. The findings suggest that exercise interventions involving supervised strength training that are group-based; increase knowledge, skills-building and social support; address financial challenges and are easily accessed; these exercise interventions will help address some of the barriers in this underserved population [46]. Recognising that black men are at an increased risk of PCa, there is a clear need to design trials to fulfil the needs of black men, or at the very least further investigate qualitatively their views on exercise interventions and barriers. A study in 2017 explored the acceptability, barriers and facilitators to lifestyle interventions in African Caribbean PCa survivors and found that a PCa diagnosis, alongside ageing, heightened men’s awareness of their health [57]. They concluded that lifestyle interventions which enhance men’s independence and are framed as helping to regain fitness and aid post-treatment recovery are appealing and acceptable to African Caribbean PCa survivors [57].

Some of the barriers to research participation have been explored in other clinical populations and include a lack of childcare, mistrust, financial constraints, relatives’ influence and beliefs, lack of communication and cultural awareness between research staff and patients [58, 59]. The under-representation of ethnic minority groups in clinical trials affects the generalisability of study findings and ultimately contributes to exacerbating bias and inequities in access to healthcare if public health policies based on such evidence are implemented. It is possible that different ethnic groups respond in distinct ways to an intervention due to variations in physiology and/or disease state. Therefore, by studying the effects of an intervention in multiple ethnic groups, we can be sure that the outcomes are applicable to all.

### Strengths and limitations

This systematic review has been conducted rigorously with regard to methodology and in line with the PRISMA guidelines. However, the main findings of this systematic review need to be considered in the context of some key limitations. For example, it is difficult to ascertain reasons for low recruitment numbers and, even when ethnicity was reported, recruitment strategy was not acknowledged in the manuscripts. Additionally, we must acknowledge that the lack of diversity may be due to the single centre studies being conducted in predominantly Caucasian areas, however, it is important that this is considered in their limitations section. More work is needed therefore to understand the barriers and facilitators of different ethnic groups to exercise research. Furthermore, recruitment strategies to ensure an inclusive sample need to be employed alongside reporting of participant ethnicities needs to be improved,

### Implications for research and practice

The findings from this systematic review demonstrate that whilst there are many studies into the benefits of exercise within PCa patients, there are clear disparities between studies on the reporting of participants’ ethnicities and overall low numbers of ethnic minority men included. This systematic review suggests that representation is achieved in a few trials. Work needs to be performed to understand why representation is lacking in PCa exercise trials in the UK and action is needed to address this. Future studies may want to explore the barriers patients from different ethnic groups face when taking part in clinical trials and any potential bias within the recruitment process. Studies should include defined strategies to recruit a representative study sample and report this within the methods section of the manuscript in accordance with NIHR INCLUDE [54] to ensure the results are applicable and representative of the patient group. Additionally, authors should clearly report the ethnicity of participants within the demographic information of manuscripts to allow the reader to properly interpret the results in the context of the patients included.

## Conclusion

This systematic review highlights that there is high heterogeneity in the reporting of participants’ ethnicity within PCa trials involving exercise. Additionally, it has also demonstrated that there are low numbers of ethnic minority men included in PCa and exercise studies in the UK and a lack of reporting of ethnicities in published papers. As such, further work is required to understand why representation is lacking within PCa exercise trials in the UK and strategies are needed to achieve representation. Future studies should seek to explore the barriers PCa patients from different ethnic groups face when taking part in clinical trials in the UK.

## Data Availability

No datasets were generated or analysed during the current study.

## References

[1] Cancer Research UK. Prostate cancer statistics. Available from: https://www.cancerresearchuk.org/health-professional/cancer-statistics/statistics-by-cancer-type/prostate-cancer#heading-Zero. Accessed 17 July 2024

[2] Wang L et al (2022) Prostate cancer incidence and mortality: global status and temporal trends in 89 countries from 2000 to 2019. Front Public Health 10:811044 35252092 10.3389/fpubh.2022.811044 PMC8888523

[3] Paterson C et al (2015) Identifying the unmet supportive care needs of men living with and beyond prostate cancer: a systematic review. Eur J Oncol Nurs 19(4):405–418 25613370 10.1016/j.ejon.2014.12.007

[4] Paterson C et al (2022) The effects of multimodal prehabilitation interventions in men affected by prostate cancer on physical, clinical and patient reported outcome measures: a systematic review. Semin Oncol Nurs 38(5):151333 35999090 10.1016/j.soncn.2022.151333

[5] Aning JJ et al (2018) Detailed analysis of patient-reported lower urinary tract symptoms and effect on quality of life after robotic radical prostatectomy. Urol Oncol 36(8):364.e15-364.e22 29891407 10.1016/j.urolonc.2018.05.017

[6] Campbell KL et al (2019) Exercise guidelines for cancer survivors: consensus statement from international multidisciplinary roundtable. Med Sci Sports Exerc 51(11):2375–2390 31626055 10.1249/MSS.0000000000002116 PMC8576825

[7] Lowder D et al (2022) Racial disparities in prostate cancer: a complex interplay between socioeconomic inequities and genomics. Cancer Lett 531:71–82 35122875 10.1016/j.canlet.2022.01.028 PMC9701576

[8] Lloyd T et al (2015) Lifetime risk of being diagnosed with, or dying from, prostate cancer by major ethnic group in England 2008–2010. BMC Med 13(1):171 26224061 10.1186/s12916-015-0405-5 PMC4520076

[9] Delon C et al (2022) Differences in cancer incidence by broad ethnic group in England, 2013–2017. Br J Cancer 126(12):1765–1773 35233092 10.1038/s41416-022-01718-5 PMC9174248

[10] Chornokur G et al (2011) Disparities at presentation, diagnosis, treatment, and survival in African American men, affected by prostate cancer. Prostate 71(9):985–997 21541975 10.1002/pros.21314 PMC3083484

[11] Ashton RE et al (2021) Supported progressive resistance exercise training to counter the adverse side effects of robot-assisted radical prostatectomy: a randomised controlled trial. Support Care Cancer 29(8):4595–4605 33483790 10.1007/s00520-021-06002-5 PMC7822752

[12] Bourke L et al (2018) Exercise training as a novel primary treatment for localised prostate cancer: a multi-site randomised controlled phase II study. Sci Rep 8(1):8374 29849032 10.1038/s41598-018-26682-0 PMC5976628

[13] Banerjee S et al (2018) Vigorous intensity aerobic interval exercise in bladder cancer patients prior to radical cystectomy: a feasibility randomised controlled trial. Support Care Cancer 26(5):1515–1523 29181804 10.1007/s00520-017-3991-2

[14] Mottet N, Cornford P, van den Bergh RCN, Briers E, Expert Patient Advocate, De Santis M, Gillessen S, Grummet J, Henry AM, van der Kwast TH, Lam TB, Mason MD, O'Hanlon S, Oprea-Lager De, Ploussard G, van der Poel HG, Rouviere O, Schoots IG, Tilki D, Wiegel T (2022) EAU guidelines on prostate cancer. Available from: https://uroweb.org/guidelines/prostate-cancer/chapter/introduction. Accessed 12 Feb 2024

[15] NICE (2014) Physical activity: exercise referral schemes - public health guideline [PH54]. Available from: https://www.nice.org.uk/guidance/ph54. Accessed 12 Feb 2024

[16] Bourke L et al (2016) Exercise for men with prostate cancer: a systematic review and meta-analysis. Eur Urol 69(4):693–703 26632144 10.1016/j.eururo.2015.10.047

[17] Andersen MF, J Midtgaard, ED Bjerre (2022) Do patients with prostate cancer benefit from exercise interventions? A systematic review and meta-analysis. Int J Environ Res Public Health 19(2): 972 10.3390/ijerph19020972 PMC8776086 35055794

[18] Hart NH, Galvão DA, Newton RU (2017) Exercise medicine for advanced prostate cancer. Curr Opin Support Palliat Care 11(3):247–257 28562375 10.1097/SPC.0000000000000276

[19] Hasenoehrl T et al (2015) The effects of resistance exercise on physical performance and health-related quality of life in prostate cancer patients: a systematic review. Support Care Cancer 23(8):2479–2497 26003426 10.1007/s00520-015-2782-x

[20] Keilani M et al (2017) Effects of resistance exercise in prostate cancer patients: a meta-analysis. Support Care Cancer 25(9):2953–2968 28600706 10.1007/s00520-017-3771-z PMC5527087

[21] Hvid T et al (2016) Effect of a 2-year home-based endurance training intervention on physiological function and PSA doubling time in prostate cancer patients. Cancer Causes Control 27(2):165–174 26573844 10.1007/s10552-015-0694-1

[22] Rencsok EM et al (2020) Diversity of enrollment in prostate cancer clinical trials: current status and future directions. Cancer Epidemiol Biomarkers Prev 29(7):1374–1380 32503813 10.1158/1055-9965.EPI-19-1616 PMC7334076

[23] Page MJ et al (2021) The PRISMA 2020 statement: an updated guideline for reporting systematic reviews. BMJ 372:n71 33782057 10.1136/bmj.n71 PMC8005924

[24] Ouzzani M, Hammady H, Fedorowicz Z, Elmagarmid A (2016) Rayyan — a web and mobile app for systematic reviews. Syst Rev 5:210 10.1186/s13643-016-0384-4 PMC5139140 27919275

[25] Alibhai SMH et al (2019) A phase II randomized controlled trial of three exercise delivery methods in men with prostate cancer on androgen deprivation therapy. BMC Cancer 19(1):2 30606137 10.1186/s12885-018-5189-5 PMC6318980

[26] Au D et al (2019) Prehabilitation and acute postoperative physical activity in patients undergoing radical prostatectomy: a secondary analysis from an RCT. Sports Med Open 5(1):18 31119491 10.1186/s40798-019-0191-2 PMC6531507

[27] Ben-Josef AM et al (2016) Yoga intervention for patients with prostate cancer undergoing external beam radiation therapy: a pilot feasibility study. Integr Cancer Ther 15(3):272–278 26590125 10.1177/1534735415617022 PMC5739183

[28] Brown M, Murphy MH, McAneney H, McBride K, Crawford F, Cole A, O'Sullivan JM, Jain S, Prue G (2023) Feasibility of home‑based exercise training during adjuvant treatment for metastatic castrate‑resistant prostate cancer patients treated with an androgen receptor pathway inhibitor (EXACT). Supportive Care Cancer 31(7):442 10.1007/s00520-023-07894-1 PMC10319656 37402060

[29] Campo RA et al (2014) Levels of fatigue and distress in senior prostate cancer survivors enrolled in a 12-week randomized controlled trial of Qigong. J Cancer Surviv 8(1):60–69 24170679 10.1007/s11764-013-0315-5 PMC3945387

[30] Chan JM et al (2020) Feasibility and acceptability of a remotely delivered, web-based behavioral intervention for men with prostate cancer: four-arm randomized controlled pilot trial. J Med Internet Res 22(12):e19238 33382378 10.2196/19238 PMC7808895

[31] Dai JY et al (2019) Vigorous physical activity is associated with lower risk of metastatic-lethal progression in prostate cancer and hypomethylation in the CRACR2A gene. Cancer Epidemiol Biomarkers Prev 28(2):258–264 30464020 10.1158/1055-9965.EPI-18-0622 PMC6363836

[32] Dalla Via J et al (2021) Musculoskeletal responses to exercise plus nutrition in men with prostate cancer on androgen deprivation: a 12-month RCT. Med Sci Sports Exerc 53(10):2054–2065 33867499 10.1249/MSS.0000000000002682

[33] Dawson JK et al (2018) Impact of resistance training on body composition and metabolic syndrome variables during androgen deprivation therapy for prostate cancer: a pilot randomized controlled trial. BMC Cancer 18(1):368 29614993 10.1186/s12885-018-4306-9 PMC5883585

[34] Evans HE et al (2021) Usability, acceptability, and safety analysis of a computer-tailored web-based exercise intervention (ExerciseGuide) for individuals with metastatic prostate cancer: multi-methods laboratory-based study. JMIR Cancer 7(3):e28370 34318759 10.2196/28370 PMC8367181

[35] Fairman CM et al (2018) Monitoring resistance exercise intensity using ratings of perceived exertion in previously untrained patients with prostate cancer undergoing androgen deprivation therapy. J Strength Cond Res 32(5):1360–1365 28557849 10.1519/JSC.0000000000001991

[36] Faithfull S et al (2021) Obesity and low levels of physical activity impact on cardiopulmonary fitness in older men after treatment for prostate cancer. Eur J Cancer Care (Engl) 30(6):e13476 34143537 10.1111/ecc.13476

[37] Friedenreich CM et al (2016) Physical activity and survival after prostate cancer. Eur Urol 70(4):576–585 26774959 10.1016/j.eururo.2015.12.032

[38] Gilbert SE et al (2016) Effects of a lifestyle intervention on endothelial function in men on long-term androgen deprivation therapy for prostate cancer. Br J Cancer 114(4):401–408 26766737 10.1038/bjc.2015.479 PMC4815775

[39] Hébert JR et al (2012) A diet, physical activity, and stress reduction intervention in men with rising prostate-specific antigen after treatment for prostate cancer. Cancer Epidemiol 36(2):e128–e136 22018935 10.1016/j.canep.2011.09.008 PMC3267863

[40] Jones LW et al (2014) Effects of nonlinear aerobic training on erectile dysfunction and cardiovascular function following radical prostatectomy for clinically localized prostate cancer. Eur Urol 65(5):852–855 24315706 10.1016/j.eururo.2013.11.009 PMC4089506

[41] Langelier DM et al (2018) Perceptions of masculinity and body image in men with prostate cancer: the role of exercise. Support Care Cancer 26(10):3379–3388 29654565 10.1007/s00520-018-4178-1

[42] Nilsen TS et al (2018) Novel methods for reporting of exercise dose and adherence: an exploratory analysis. Med Sci Sports Exerc 50(6):1134–1141 29315168 10.1249/MSS.0000000000001545 PMC5953772

[43] Sajid S et al (2016) Novel physical activity interventions for older patients with prostate cancer on hormone therapy: a pilot randomized study. J Geriatr Oncol 7(2):71–80 26916611 10.1016/j.jgo.2016.02.002 PMC4818675

[44] Santa Mina D et al (2013) Exercise effects on adipokines and the IGF axis in men with prostate cancer treated with androgen deprivation: a randomized study. Can Urol Assoc J 7(11–12):E692–E698 24282459 10.5489/cuaj.235 PMC3840521

[45] Santa Mina D et al (2018) Prehabilitation for radical prostatectomy: a multicentre randomized controlled trial. Surg Oncol 27(2):289–298 29937184 10.1016/j.suronc.2018.05.010

[46] Stolley MR et al (2020) Exploring health behaviors, quality of life, and support needs in African-American prostate cancer survivors: a pilot study to support future interventions. Support Care Cancer 28(7):3135–3143 31705377 10.1007/s00520-019-05092-6

[47] Taaffe DR et al (2017) Effects of different exercise modalities on fatigue in prostate cancer patients undergoing androgen deprivation therapy: a year-long randomised controlled trial. Eur Urol 72(2):293–299 28249801 10.1016/j.eururo.2017.02.019

[48] Trinh L et al (2018) RiseTx: testing the feasibility of a web application for reducing sedentary behavior among prostate cancer survivors receiving androgen deprivation therapy. Int J Behav Nutr Phys Act 15(1):49 29880049 10.1186/s12966-018-0686-0 PMC5992665

[49] Wang Y et al (2017) Recreational physical activity in relation to prostate cancer-specific mortality among men with nonmetastatic prostate cancer. Eur Urol 72(6):931–939 28711382 10.1016/j.eururo.2017.06.037

[50] Winters-Stone KM et al (2016) Benefits of partnered strength training for prostate cancer survivors and spouses: results from a randomized controlled trial of the Exercising Together project. J Cancer Surviv 10(4):633–644 26715587 10.1007/s11764-015-0509-0

[51] Wolin KY et al (2010) Risk of urinary incontinence following prostatectomy: the role of physical activity and obesity. J Urol 183(2):629–633 20018324 10.1016/j.juro.2009.09.082 PMC3034651

[52] Higgins JPT et al (2011) The Cochrane Collaboration’s tool for assessing risk of bias in randomised trials. BMJ 343:d5928 22008217 10.1136/bmj.d5928 PMC3196245

[53] Julian PT Higgins, JT Jacqueline Chandler, Miranda Cumpston, Tianjing Li, Matthew J Page, Vivian A Welch (2019) Cochrane Handbook for systematic reviews of interventions, 2nd ed, Wiley-Blackwell 10.1002/14651858.ED000142 PMC10284251 31643080

[54] NIHR (2020) Improving inclusion of under-served groups in clinical research: guidance from the NIHR-INCLUDE project. UK: NIHR. 14th March 2023]. Available from: www.nihr.ac.uk/documents/improving-inclusion-of-under-served-groups-in-clinical-research-guidance-from-include-project/25435. Accessed 12 Feb 2024

[55] Jenson E, Jones N, Orozco K, Medina L, Peery M, Bolender B, Battle K (2022) Measuring racial and ethnic diversity for the 2020 census. Available from: https://www.census.gov/newsroom/blogs/random-samplings/2021/08/measuring-racial-ethnic-diversity-2020-census.html. Accessed 23 July 2024

[56] UK Government (N.D) List of ethnic groups. Available from: https://www.ethnicity-facts-figures.service.gov.uk/style-guide/ethnic-groups/. Accessed 23 July 2024

[57] Er V et al (2017) Barriers and facilitators to healthy lifestyle and acceptability of a dietary and physical activity intervention among African Caribbean prostate cancer survivors in the UK: a qualitative study. BMJ Open 7(10):e017217 29038181 10.1136/bmjopen-2017-017217 PMC5652511

[58] Waheed W et al (2015) Overcoming barriers to recruiting ethnic minorities to mental health research: a typology of recruitment strategies. BMC Psychiatry 15(1):101 25934297 10.1186/s12888-015-0484-z PMC4436137

[59] Duma N et al (2018) Representation of minorities and women in oncology clinical trials: review of the past 14 years. J Oncol Pract 14(1):e1–e10 29099678 10.1200/JOP.2017.025288

